# Examining the relationship between stressful life events and overgeneral autobiographical memory in adolescents at high familial risk of depression

**DOI:** 10.1080/09658211.2018.1508591

**Published:** 2018-08-18

**Authors:** Naomi Warne, Stephan Collishaw, Frances Rice

**Affiliations:** MRC Centre for Neuropsychiatric Genetics and Genomics, Division of Psychological Medicine and Clinical Neurosciences, Cardiff University, Cardiff, UK

**Keywords:** Autobiographical memory, stressful life events, adolescence, depression

## Abstract

Difficulty remembering specific events from the personal past, known as overgeneral autobiographical memory (AM), may be a marker of vulnerability to adolescent depression but little is known about how overgeneral AM arises in this age group. Stressful life events (SLEs) are strongly implicated in the onset of depression and are considered important in theoretical work on AM. We investigated whether exposure to lifetime and recent SLEs contributed to the development of overgeneral AM in a sample of adolescents at high familial risk of depression (*n* = 257) and examined the effects of gender and memory valence. Whether AM mediated the relationship between SLEs and MDD was also assessed. Exposure to a higher number of lifetime SLEs was associated with an increase in *specific* AMs. Associations of recent SLEs with AM differed by gender. For girls, more recent SLEs were associated with more overgeneral AMs. For boys, more recent SLEs were associated with fewer overgeneral AMs and more specific AMs. AM did not mediate the relationship between SLEs and subsequent DSM-IV depressive symptom count. Results suggest a complex relationship between AM and SLEs and that overgeneral AM and SLEs may have independent effects on future depression.

Major Depressive Disorder (MDD) is a common, chronic condition and is now the leading cause of disability worldwide (Friedrich, 2017; World Health Organization, 2017). During adolescence the incidence of MDD increases markedly (Kessler et al., 2005) and depression that begins early is associated with particularly poor outcomes and a chronic, long-term course of symptoms (Dunn & Goodyer, 2006; Patton et al., 2014). MDD has a complex aetiology encompassing both environmental and genetic risk factors (Sullivan, Neale, & Kendler, 2000; Thapar, Collishaw, Pine, & Thapar, 2012). The most common and potent risk factor for early-onset depression is having a parent with MDD which increases the risk of depression to offspring 3 to 4 fold (Rice, Harold, & Thapar, 2002; Weissman et al., 2006). Investigating the processes that underlie risk for depression in this high risk group is therefore important for prevention and early intervention.

One factor that appears to increase risk for MDD is overgeneral autobiographical memory. There is good evidence that memory is altered in the depressed state. In particular, depressed individuals have difficulty in remembering details of events from their personal past, i.e., autobiographical memory (AM). When asked to recall a specific event, individuals with MDD tend to retrieve fewer specific instances (reduced AM specificity) and more “overgeneral” memories covering extended time periods or repeated events (increased overgeneral AM) (Liu, Li, Xiao, Yang, & Jiang, 2013; Williams et al., 2007). AM is important as it affects a number of processes thought to be disrupted in MDD. For instance, recalling positive memories can act as a useful mood regulatory strategy (Joormann & Siemer, 2004; Joormann, Siemer, & Gotlib, 2007; Ramirez et al., 2015) and memories of the past can affect the ability to solve social problems (Goddard, Dritschel, & Burton, 1996, 1997), which may, in turn, influence mood. Finally, AMs are closely associated with sense of self, validating and supporting self-perceptions (Conway & Pleydell-Pearce, 2000), so are important for maintaining self-esteem. Deficits in accessing specific details of AMs can therefore result in reductions in effective emotion regulation and self-reflection, which may increase the risk of MDD. Indeed, overgeneral AM and reduced AM specificity have been found to predict subsequent depressive symptoms in adults (Sumner, Griffith, & Mineka, 2010). Importantly, work in an adolescent sample has found that overgeneral AM for negative cues is prospectively associated with new-onset MDD and higher depressive symptoms (Rawal & Rice, 2012a). Preliminary evidence also suggests that interventions aimed at increasing specific AMs can improve depressive symptoms (Hitchcock, Werner-Seidler, Blackwell, & Dalgleish, 2017; Koehler et al., 2015; Neshat-Doost et al., 2013).

Despite the involvement of overgeneral AM in depression, little is understood about how the phenomenon develops. One prominent theory proposes that overgeneral AM arises through capture and rumination, functional avoidance and impaired executive control (CaR-FA-X model; Williams et al., 2007). This theory proposes that individuals retrieving a specific memory will use a generative retrieval process which starts at a broad, generic, level before moving down towards event-specific knowledge. If this hierarchical search is interrupted then an overgeneral AM may be retrieved instead of a more specific memory. It is proposed that capture and rumination, can interrupt this generative retrieval process as emotional cues activate general self-relevant information resulting in task irrelevant rumination. The functional avoidance mechanism is posited to occur when individuals passively avoid recalling specific details of AMs as a way of reducing emotional distress. Overgeneral AM is hypothesised to develop early in life initially as an emotion regulatory strategy to deal with memories of traumatic or stressful life events (SLEs) but then becomes generalised to other memories through reinforcement (Hermans et al., 2008; Williams et al., 2007). The final mechanism, impaired executive functioning, is the inability to perform this hierarchical search due to lack of, or reduced, executive resources. Support has been found for rumination and impaired executive control contributing to overgeneral AM (Rawal & Rice, 2012b; Sumner, 2012), but the role of functional avoidance is less clear (Sumner, 2012).

A considerable amount of research has explored functional avoidance indirectly by assessing overgeneral AM in individuals who have experienced traumatic events (such as abuse, sudden serious illness/injury, combat, motor vehicle accidents, assault and rape) (Moore & Zoellner, 2007; Ono, Devilly, & Shum, 2015). Evidence has suggested that trauma alone is not sufficient for overgeneral AM to develop (Moore & Zoellner, 2007; Williams et al., 2007) and research on whether this relationship could be moderated by factors such as age (e.g., childhood) and type of trauma (e.g., sexual abuse) is inconsistent (Crane & Duggan, 2009; Johnson, Greenhoot, Glisky, & McCloskey, 2005; Moore & Zoellner, 2007; Sumner, 2012; Valentino, Toth, & Cicchetti, 2009). Although the majority of these studies have only indirectly assessed functional avoidance, more direct evidence in non-clinical samples has suggested that avoiding specific details of negative memories can act as an effective emotion regulation strategy to specific stressors (Anderson, Goddard, & Powell, 2010; Hermans et al., 2008). Overgeneral AM and reduced AM specificity are also associated with strategies such as thought suppression and dissociation (Schonfeld & Ehlers, 2006; Williams et al., 2007), thereby highlighting the role of overgeneral AM as a cognitive avoidance strategy. Thought suppression and dissociation are also implicated in disorders linked with increased overgeneral AM such as depression and PTSD (Lyssenko et al., 2018; Wenzlaff & Wegner, 2000).

Despite the evidence for an association between trauma and overgeneral AM (Moore & Zoellner, 2007; Ono et al., 2015), there has been less consideration of the association between overgeneral AM and SLEs that are not overtly traumatic, such as disappointment and loss events despite their importance in the aetiology of depression (Goodyer, Cooper, Vize, & Ashby, 1993; NICE, 2005). Studies that have explored SLEs and overgeneral AM tend to focus on recent specific stressors in adult samples (e.g., failing first university exam (Hermans et al., 2008) and daily hassles (Anderson et al., 2010)). Given that the functional avoidance mechanism likely develops early in life in response to a wide range of stressors, more work is necessary in younger samples looking at a range of lifetime and recent SLEs. Lifetime exposures to trauma and SLEs are important as rather than being time-limited, they can act as risk processes that occur over time and can have long-lasting effects (Chapman et al., 2004; Repetti, Taylor, & Seeman, 2002; Rutter & Sroufe, 2000). However, research into recent SLEs is also warranted as they are common and are thought to play a causal role in MDD (Kendler & Gardner, 2010; Kendler, Karkowski, & Prescott, 1999), including in samples at high familial risk of depression (Goodyer et al., 1993; Rice et al., 2017). Although both recent and lifetime SLEs play a role in depression, stronger effects are seen between SLEs and MDD when they occur in the same developmental period (Shanahan, Copeland, Costello, & Angold, 2011); thus different effects may be seen between SLEs and overgeneral AM depending on recency of the events.

Adolescence may be an important period for the development of overgeneral AM as it is a key period for increases in SLEs, biological sensitivity to stress, and rates of MDD (Ge, Conger, & Elder, 2001; Lupien, McEwen, Gunnar, & Heim, 2009; Maughan, Collishaw, & Stringaris, 2013). Gender differences in SLEs and depression also begin to emerge in adolescence. Adolescent girls display an increase in social stress in comparison to adolescent boys and this heightened sensitivity to stress is thought to contribute to the increase in depressive symptomatology (Rice, Harold, & Thapar, 2003; Shih, Eberhart, Hammen, & Brennan, 2006; Thapar et al., 2012). There may therefore be gender differences in the effect of SLEs on overgeneral AM in adolescence but this has not been previously investigated. As around 40% of depressed parents’ offspring develop MDD by young adulthood (Weissman et al., 2006; Weissman et al., 2016), studying the relationship between SLEs and overgeneral AM in adolescent samples at high familial risk of depression can potentially help elucidate whether targeting AM is a useful prevention strategy for those at risk.

Although SLEs have classically been seen as risk factors for psychopathology, moderate amounts or particular types of stress may be adaptive and promote development of coping mechanisms. It has been highlighted that children in supportive, enriching environments and children experiencing high levels of adversity or chronic stress both exhibit heightened sensitivity to stress whereas those experiencing moderate stressors do not display this heightened sensitivity (Boyce & Ellis, 2005; Ellis & Boyce, 2008). Several instances of such curvilinear relationships between SLEs and mental health have been reported (Höltge, Mc Gee, & Thoma, 2018; McLafferty et al., 2018; Shapero et al., 2015); however nature of stressor (e.g., exam stress versus death of mother) is also likely to be important. Given the previous literature on curvilinear relationships between SLEs and mental health it is also important to assess non-linear relationships between SLEs and AM.

Valence of memory may also play a role in the relationship between SLEs and overgeneral AM. Functional avoidance is thought to develop initially for negative memories and then generalise to more positive memories (Williams et al., 2007). Previous research has also found associations between adolescent depression scores and overgeneral AMs for negative cues but not positive cues (Rawal & Rice, 2012a; Woody, Burkhouse, & Gibb, 2015). Consequently, SLEs may be more strongly associated with negative overgeneral AMs than positive overgeneral AMs in adolescence. Previous studies have primarily focused on valence of cue words under the assumption that cue valence and memory content valence are the same. However, this is not always the case and cue-memory valence similarity can vary depending on MDD status (Young, Erickson, & Drevets, 2012). Consequently, separate analyses for cue valence and memory valence have been advised (Lemogne, Limosin, & Fossati, 2013). It is therefore important to consider both cue and memory valence when investigating the relationship between SLEs and overgeneral AM. Although self-ratings would presumably be the gold standard, researcher-rated valence has been used in cases where self-ratings are not available (Chen et al., 2015; Meyer, Karl, & Flor, 2015; Sansom-Daly, Bryant, Cohn, & Wakefield, 2014; Schulkind, Rahhal, Klein, & Lacher, 2012).

The aim of the current study was to explore the relationship between SLEs and AM in a prospective longitudinal sample of adolescents with depressed parents (The Early Prediction of Adolescent Depression (EPAD) study) (Mars et al., 2012; Mars et al., 2013). Previous work with this sample found that overgeneral AM for negative cue words predicts subsequent DSM-IV MDD and depressive symptom count (Rawal & Rice, 2012a). In order to better understand the developmental pathways underlying overgeneral AM as a risk factor for depression, the current study set out to examine: 1) the relationship between SLEs and AM. We particularly focused on examining the relationship between SLEs and overgeneral AM given that overgeneral AM is the aspect of autobiographical memory most strongly associated with vulnerability for subsequent depression in this sample. We examined AM specificity as a secondary outcome as this is consistent with previous literature (Liu et al., 2013; Moore & Zoellner, 2007; Williams et al., 2007) and improving specific AMs has been implicated as a potential treatment strategy for depression (Hitchcock et al., 2017; Koehler et al., 2015; Neshat Doost et al., 2014). Both linear and non-linear relationships were assessed. We additionally examined the role of a) recency of exposure to SLEs (whether the association differed for lifetime versus recent exposure to SLEs); b) gender; and c) emotional valence (cue word and memory content valence). It was hypothesised that SLEs (lifetime and recent) would be associated with more overgeneral AMs and fewer specific AMs (Williams et al., 2007). Stronger relationships for females were anticipated given that the predictive relationship between overgeneral AM and depression is stronger in girls (Rawal & Rice, 2012a) and girls experience a greater number of social stressors in adolescence (Hamilton, Stange, Abramson, & Alloy, 2015; Shih et al., 2006). Based on the functional avoidance theory (Williams et al., 2007), it was anticipated that SLEs would have a stronger relationship with negative overgeneral AMs and overgeneral AMs generated from negative cue words given that negative cue words are more likely to result in negative memories. 2) We also set out to test if AM mediated the relationship between SLEs and subsequent DSM-IV depressive symptom count. We were particularly interested in testing the hypothesis that overgeneral AM for negative cues mediated the relationship between SLEs and depression given the previously reported association between overgeneral AM for negative material and depressive disorder in this sample. Sensitivity analyses checked the role of event severity and previous episodes of MDD.

## Methods

### Sample and design

Data were collected during three waves of the EPAD study (Mars et al., 2012; Mars et al., 2013) via interviews at the participants’ homes and questionnaires. The initial sample included 337 children and adolescents (140 male, 197 female, aged 9–17 years at baseline, mean age 12 years) with a parent with recurrent DSM-IV MDD (American Psychiatric Association, 1994). Parents and children reported on SLEs at baseline (Wave 1, *n* = 279) and a second time point (Wave 2, *n* = 261). AM was assessed at the second time point (Wave 2, *n* = 257, 155 female, 102 male) which occurred on average 16 months after baseline. See Table 1 for descriptive information on this sample.

**Table 1.** Descriptive information, gender differences and associations between SLEs, AM, depressive symptoms, covariates and descriptive variables.

	1	2	3	4	5	6	7	8	9	10	11	12	13	14	15	16
1. Age																
2. IQ	−**.126**															
3. Working memory	−.014	**.667**														
4. Economic disadvantage	.067	−**.232**	−.094													
5. W2 depressive symptoms (MFQ)	.092	−.075	−.071	**.177**												
6. W3 depressive symptoms (MFQ)	.079	−.013	.029	.114	**.635**											
7. W2 DSM-IV depressive symptom count (CAPA)	**.234**	−**.161**	−.124	**.160**	**.686**	**.487**										
8. W3 DSM-IV depressive symptom count (CAPA)	**.188**	−.099	−.047	**.165**	**.509**	**.691**	**.619**									
9. SLEs (lifetime)	**.280**	−**.172**	−**.211**	**.230**	**.215**	.106	**.222**	**.174**								
10. SLEs (recent)	.092	−.046	−.025	−.015	**.538**	**.352**	**.461**	**.360**	**.148**							
11. Overgeneral AM (total)	−.092	.055	−.002	.022	**.173**	**.160**	.067	**.144**	−.060	.098						
12. Specific AM (total)	.108	**.192**	.107	−.063	−.100	−.084	−.055	−.094	.126	−.069	−**.503**					
13. Overgeneral AM (positive cues)	−.086	.033	−.021	−.051	.067	.086	.015	.041	−.100	.049	**.818**	−**.438**				
14. Overgeneral AM (negative cues)	−.065	.057	.018	.088	**.220**	**.179**	.093	**.192**	.003	.114	**.811**	−**.382**	**.327**			
15. Specific AM (positive cues)	.110	**.209**	**.147**	−.011	−.106	−.078	−.053	−.070	**.166**	−.111	−**.461**	**.875**	−**.529**	−**.219**		
16. Specific AM (negative cues)	.082	**.131**	.043	−.098	−.070	−.070	−.044	−.093	.058	−.011	−**.426**	**.885**	−**.247**	−**.448**	**.550**	
Mean or %	13.735	96.534	97.516	30.3%	**16.814**	**16.037**	**1.905**	**1.798**	3.771	**2.822**	2.039	6.817	1.109	0.930	3.518	3.300
SD or *n*	2.019	11.728	13.762	77/254	13.639	13.778	1.960	1.919	1.937	2.099	1.849	2.917	1.144	1.126	1.623	1.691
Range	10–18	69–131	56–135		0–57	0–63	0–9	0–9	0–9	0–9	0–9	0–12	0–5	0–6	0–6	0–6

AM = autobiographical memory; CAPA = Child and Adolescent Psychiatric Assessment; IQ = Intelligence Quotient; MFQ = Mood and Feelings Questionnaire; SLEs = stressful life events; W2 = Wave 2; W3 = Wave 3. Significant correlations and significant gender differences in means from t-tests at *p* < .05 indicated in bold. Correlations by gender are displayed in Supplemental Table 2.

### Measures

#### Stressful life events

Life events were assessed using a modified version of the Life Events Checklist (Johnson & McCutcheon, 1980). As the focus of the study was on negative events, only events that were unambiguously negative were considered SLEs. Two versions were completed by parents and children; one assessing the occurrence of more severe lifetime SLEs (at baseline, Wave 1) and one assessing the occurrence of recent SLEs in the past 12 months (at Wave 2). Lifetime SLEs included 12 items, whereas recent SLEs included 21 items (see Supplemental Material, Table 1). It was judged that these SLEs could be recalled retrospectively with accuracy (Brewin, Andrews, & Gotlib, 1993). The reporting time frames for lifetime SLEs (Wave 1) and recent SLEs (Wave 2) did not overlap as the second assessment occurred on average 16 months later. Parents and children also rated the impact of each life event on a 5 point scale (1 Very pleasant, 2 A bit pleasant, 3 No effect/neutral, 4 A bit unpleasant, 5 Very unpleasant). Parent and child reports were combined for each item so that if one respondent reported a life event then it was considered present (i.e., an OR rule for each item) as multiple informants are considered to provide valid information with discrepancies reflecting omissions and differential knowledge of the event (Gest, Reed, & Masten, 1999). Items were summed to generate total number of lifetime SLEs and total number of recent SLEs.

#### Autobiographical memory test

Participants completed the Autobiographical Memory Test (AMT) (Williams & Broadbent, 1986) as previously reported (Rawal & Rice, 2012a, 2012b). Participants were asked to recall a specific personal memory for 12 emotional cue words (6 positive, 6 negative from one of two word sets). Participants initially completed three practice trials and received feedback with further prompting to be specific for any nonspecific responses. During the main trials each of the 12 cue words was read aloud and participants were given 30 seconds in which to respond. If a specific memory was not retrieved participants were verbally prompted (e.g., “Can you think of a specific time?”) and if no memory was retrieved an omission was recorded.

*Rating AMs as specific or overgeneral:* Responses were transcribed and coded as either: specific (memories specific to time and place e.g., “the day we got our dog from the rescue shelter”), extended (spanning longer than one day e.g., “when we went on holiday with our dog”), categoric (repeated events of a similar nature e.g., “when walking my dog”) or semantic associates (related to cue word but not a memory e.g., “my dog”). Raters were blind to history of SLEs. There was high inter-rater reliability from two independent raters who coded the responses of 45 participants (17.51% of the full sample; average agreement *κ* = 0.93) (Rawal & Rice, 2012a, 2012b). Number of overgeneral AMs (categoric plus extended responses) was the primary outcome. Number of specific AMs was a secondary outcome. Additional outcomes related to valence of the cue word, namely overgeneral responses to positive cues (e.g., happy, sunny), overgeneral responses to negative cues (e.g., angry, mistake), specific responses to positive cues, and specific responses to negative cues (each out of 6).

*Rating AM content valence:* Content of the memories was also coded for valence by raters blind to SLE history. All memories were coded as negative, neutral or positive by the first author and responses from 45 (17.51%) participants were also coded by the last author. Inter-rater reliability was good: average weighted kappa across all cues was 0.65 (SD 0.26) and average percentage agreement was 86.15% (SD 8.80) which is similar to previously reported rates of agreement for memory valence (e.g., Schulkind et al., 2012). Disagreements were discussed and coded according to consensus between raters. Low rates of neutral AMs precluded analysis. The following outcome variables were therefore calculated: overgeneral positive memories, overgeneral negative memories, specific positive memories and specific negative memories.

#### Covariates, psychopathology at follow-up, and descriptive variables

Analyses adjusted for a number of factors known to be associated with overgeneral AM: IQ, age, gender and depressive symptoms (Park, Goodyer, & Teasdale, 2002; Pillemer, Wink, DiDonato, & Sanborn, 2003; Reese, Haden, & Fivush, 1996; Williams et al., 2007). *Child IQ* was assessed at interview (Wave 1) using 10 subscales on the Wechsler Intelligence Scale for Children (WISC, Wechsler, 2003). WISC scores were standardised so the mean in the total sample was 100, in line with population norm scoring. *Child current depressive symptoms* contemporaneous with performance of the AMT (Wave 2) were assessed using the Mood and Feelings Questionnaire (MFQ, Angold & Costello, 1987), which comprised 34 items and covered the previous 3 months. The MFQ was chosen as a covariate as it allowed control for a full range of depressive symptoms. The MFQ has good reliability and validity, and is considered a valid screening tool for depression (Angold, 1989; Daviss et al., 2006; Kent, Vostanis, & Feehan, 1997). Index parent and child rated depressive symptoms were combined using an OR rule for each item to provide a reliable estimate of children’s current depression (Cronbach’s *α* = 0.954). Length of follow-up was not associated with recent SLEs (*r* = −.007, 95% CI = −.139, .125, *p* = .922), so was not added as a covariate in analyses.

*Child DSM-IV depressive symptom count at follow-up* was measured at Wave 3 using combined parent and child reports (OR rule) on the on the Child and Adolescent Psychiatric Assessment (CAPA, Angold & Costello, 2000). This is a semi-structured diagnostic interview assessing psychiatric symptoms and impairment over the previous 3 months. CAPA depressive symptoms provide a measure of depression more closely aligned with the diagnosis of Major Depressive Disorder (American Psychiatric Association, 1994) as it includes only symptoms that are associated with incapacity and so are impairing if endorsed. MDD symptoms were summed to form total current DSM-IV depressive symptom count (possible range 0–9). DSM-IV depressive symptom count was used as the depression outcome as it is more closely aligned with clinical diagnostic criteria for MDD.

*Child current DSM-IV depressive symptom count* was used as a descriptive variable. It was measured as the number of DSM-IV MDD symptoms at Wave 2 from parent and child reports on the CAPA. *Child questionnaire depressive symptoms at follow-up* on the MFQ (Wave 3, Cronbach’s *α* = 0.957) were also included for descriptive analysis. Total number of symptoms was used for the CAPA and the MFQ. *Child working memory* was assessed using the working memory subscale on the WISC (Wechsler, 2003). *Economic disadvantage* was a dichotomous variable defined as whether participants met the international definition for poverty, i.e., ≤60% of the median income (Gordon, 2006), in this sample: Wave 1 parent-reported household income of ≤£20,000 (Rice et al., 2017).

### Procedure

Ethical approval was obtained by the Multi-Centre Research Ethics Committee for Wales and parental consent and child assent were obtained. SLEs were assessed via questionnaires at Waves 1 and 2; AMT data was collected at interview at Wave 2; depressive symptoms and DSM-IV depressive symptoms counts were assessed at Waves 2 and 3 via questionnaires and interviews.

### Statistical analysis

SPSS version 23.0 was used for analyses. Descriptive analyses included Pearson’s correlation coefficients and t-tests. To examine the first research question, associations between SLEs and AM were examined using hierarchical multiple regression models with standardised independent variables. Total number of lifetime and recent SLEs were examined as predictors in separate models. The primary outcome variable was number of overgeneral AMs. The secondary outcome variable was number of specific AMs. In regression models, the effect of covariates (gender, IQ, age, contemporaneous depressive symptoms (MFQ) at Wave 2) on AM was assessed in Step 1 and Step 2 examined the main effect of SLEs. A SLE by gender interaction term was introduced in Step 3 to determine whether the relationship between SLEs and overgeneral AM was moderated by gender (coded males 0, females 1). Non-linear main effects of SLEs on AM were tested by including a quadratic term.

The effects of gender were investigated using gender interactions as described above. Where significant gender interactions were observed, they were followed up by simple slopes analysis for males and females (Dawson, 2014).

Where a main effect of SLEs on AM was observed, follow-up analyses examined the influences of both cue word and memory content valence (positive, negative) separately. To investigate the difference in the relative magnitude of associations by valence 95% confidence intervals (CIs) of *β* were estimated using bias corrected bootstrapping (1,000 re-samples) and compared (Cumming, 2009). Where moderation of gender was evident, separate regression models for males and females were performed for each cue and memory valence. Overlapping CIs were compared (as above) to assess differences in relative magnitude of valence associations.

To test if AM mediated the relationship between SLEs and subsequent DSM-IV depressive symptom count, mediation analyses were performed using the PROCESS version 3.0 SPSS macro (Hayes, 2017). Variables were mean centred and 95% confidence intervals (CIs) were generated using 5,000 bootstrap samples. As described previously, overgeneral AM to negative cues was first investigated given previous evidence that this predicts subsequent DSM-IV depressive symptom count and disorder in this sample. Initial mediation models investigated whether overgeneral AM to negative cues (M) mediated the relationships between SLEs (X) and prospective depression symptoms at Wave 3 (Y) when controlling for current depressive symptoms on the MFQ (Wave 2). DSM-IV depressive symptom count was used as the outcome (Y) as the stringent nature of the CAPA interview provides a more pathological measure than the MFQ. Overgeneral AM to negative cues was considered to mediate the relationship between SLEs and MDD when CIs for the indirect effect did not cross zero. Although overgeneral AM for negative cues was the hypothesised mediator, for completeness mediation models also examined the mediation effects of the additional AM measures (overgeneral AM to positive cues, specific AM to negative cues, specific AM to positive cues). Finally, moderated mediation was performed using PROCESS, model 8, in SPSS (Hayes, 2015) to assess whether mediation differed by gender.

A sensitivity check examined the effect of severity of SLEs where separate analyses were run for more and less severe lifetime SLEs (see Supplemental Material, Table 1). More severe SLEs were identified as rarer events that encompassed death or serious illness of a first-degree relative, self or close friend and events that were Adverse Childhood Experiences (Chapman et al., 2004). Additional sensitivity checks were performed to ensure results were attributable to SLEs rather than a previous depressive episode. Regression models examining effects of SLEs on AM were repeated excluding individuals with a previous episode of depression (i.e., research diagnosis of MDD on the CAPA at Wave 1; *n* = 6).

## Results

### Descriptive analysis

Descriptive information on the sample including gender differences and associations between study variables is illustrated in Table 1. Details on individual SLEs with their frequency and impact is presented in Supplemental Material, Table 1. Table 1 shows that, as expected, girls experienced a greater number of recent SLEs (*t*(217) = 2.225, *p* = .027) and more depressive symptoms than boys (Wave 2 questionnaire depressive symptoms *t*(240) = 2.622, *p* = .009; Wave 3 questionnaire depressive symptoms *t*(241) = 3.298, *p* = .001; Wave 2 DSM-IV depressive symptom count *t*(251) = 2.487, *p* = .014; Wave 3 DSM-IV depressive symptom count *t*(241) = 3.119, *p* = .002). Participants typically produced more specific AMs than overgeneral AMs. Overgeneral AM and specific AM were associated with different covariates. Thus, participants with higher IQ (*r* = .192, 95% CI = .070, .307, *p* = .002) retrieved more specific AMs. In contrast, overgeneral AM was correlated with depression symptoms: questionnaire depressive symptoms at Wave 2 (*r* = .173, 95% CI = .048, .292, *p* = .007) and Wave 3 (*r* = .160, 95% CI = .035, .280, *p* = .013) and DSM-IV depressive symptom count at Wave 3 (*r* = .144, 95% CI = .018, .265, *p* = .024). Lifetime SLEs were associated with lower IQ (*r* = −.172, 95% CI = −.296, −.042, *p* = .01), lower working memory (*r* = −.211, 95% CI = −.332, −.082, *p* = .001), economic disadvantage (*r* = .230, 95% CI = .102, .350, *p* < .001) and (older) child age (*r* = .280, 95% CI = .155, .395, *p* < .001). With the exception of lifetime SLEs and Wave 3 questionnaire depressive symptoms, lifetime SLEs and recent SLEs were associated with all depression indices (lifetime: rs > .174, ps < .011; recent: rs > .352, ps < .001).

### Relationships between lifetime stressful life events and autobiographical memory

#### Overgeneral autobiographical memory

Results of hierarchical multiple regression analyses examining the association between lifetime SLEs and overgeneral AM are presented in Table 2. There was no main effect of lifetime SLEs on overgeneral AMs (*β* = −.061, *B* (95% CI) = −.111 (−.367, .146), *p* = .396) and no lifetime SLEs by gender interaction (*β* = −.013, *B* (95% CI) = −.030 (−.517, .456), *p* = .902). There was no evidence of non-linear effects of SLEs on overgeneral AM as the quadratic term was non-significant.

**Table 2.** Regression models investigating the effect of lifetime SLEs on number of overgeneral AMs and specific AMs.

	Overgeneral AM (*n* = 213)						Specific AM (*n* = 213)					
	Model change			Coefficients			Model change			Coefficients		
	*R*^2^	*p*	*f^2^*	*β*	*B* (95% CI)	*p*	*R*^2^	*p*	*f^2^*	*β*	*B* (95% CI)	*p*
**Step 1: Covariates**	**0.057**	**.015**	**0.061**				**0.087**	**.001**	**0.095**			** **
Full scale IQ				0.019	0.037 (−0.229, 0.304)	.782				0.245	0.761 (0.341, 1.180)	**<.001**
Age				−0.167	−0.303 (−0.549, −0.058)	**.016**				0.174	0.508 (0.122, 0.893)	**.010**
MFQ depressive symptoms				0.186	0.355 (0.093, 0.616)	**.008**				−0.083	−0.252 (−0.663, 0.159)	.228
Gender				−0.011	−0.040 (−0.543, 0.463)	.876				−0.002	−0.012 (−0.803, 0.779)	.976
**Step 2: Main effect SLEs**	Δ0.003	.396	0.003				**Δ0.028**	**.012**	**0.031**			** **
Lifetime SLEs				−0.061	−0.111 (−0.367, 0.146)	.396				0.178	0.512 (0.114, 0.910)	**.012**
**Step 3: Moderation by gender**	Δ<0.001	.902	<0.001				Δ0.002	.554	0.002			
Lifetime SLEs x Gender				−0.013	−0.030 (−0.517, 0.456)	.902				0.059	0.227 (−0.527, 0.981)	.554

AM = Autobiographical Memory; CI = Confidence Interval; IQ = Intelligence Quotient; MFQ = Mood and Feelings Questionnaire (Wave 2); SLEs = stressful life events. Results significant at *p* < .05 are indicated in bold. The quadratic term (lifetime SLEs x lifetime SLEs) was not significantly associated with overgeneral AM (*β* = −0.447, *B* (95% CI) = −0.826 (−1.694, 0.042), *R*^2^ change = 0.016, *f*^2^ = 0.017, *p* = .062) or specific AM (*β* = 0.280, *B* (95% CI) = 0.825 (−0.528, 2.178), *R*^2^ change = 0.006, *f*^2^ = 0.007, *p* = .230). Lifetime SLEs were significantly associated with specific AMs to positive cues (*β* = 0.220, bootstrapped 95% CI = 0.089, 0.359, *p* = .002), but not specific AMs to negative cues (*β* = 0.093, bootstrapped 95% CI = −0.047, 0.234, *p* = .203). Similarly, lifetime SLEs were significantly associated with specific positive memories (*β* = 0.241, bootstrapped 95% CI = 0.103, 0.381, *p* = .001) but not specific negative memories (*β* = 0.125, bootstrapped 95% CI = −0.009, 0.266, *p* = .085).

#### Specific autobiographical memory

More lifetime SLEs were associated with a greater number of specific AMs (*β* = .178, *B* (95% CI) = .512 (.114, .910), *p* = .012). There was no moderation by gender and there was no association between the quadratic term and specific AMs (Table 2).

*Cue word valence and memory content valence.* Follow-up analyses of the main effect of lifetime SLEs on AM specificity by cue word showed a significant relationship for specific AMs to positive cues (*β* = .220, *B* (95% CI) = .357 (.137, .577), *p* = .002) but not negative cues (*β* = .093, *B* (95% CI) = .155 (−.084, .394), *p* = .203). The difference in *β*s for positive and negative cues did not differ significantly as the upper bound CI of specific AM to negative cues exceeded 50% of the CI for specific AM to positive cues (*p* > .05; Table 2).

Similarly, lifetime SLEs were significantly associated with specific positive memories (*β* = .241, *B* (95% CI) = .391 (.169, .613), *p* = .001) but not specific negative memories (*β* = .125, *B* (95% CI) = .205 (−.029, .439) *p* = .085). The effect of lifetime SLEs on specific AM did not differ significantly by memory content valence (Table 2).

### Relationships between recent stressful life events and autobiographical memory

#### Overgeneral autobiographical memory

When the relationship between recent SLEs and overgeneral AM was examined (Table 3), there was no main or quadratic effect of recent SLEs. A significant SLE by gender interaction was evident (*β* = .349, *B* (95% CI) = .882 (.340, 1.423), *p* = .002). Simple slopes analysis showed effects in opposing directions for males (*t* = −2.233, *p* = .027) and females (*t* = 1.996, *p* = .047). Analyses for recent SLEs and positive/negative overgeneral AM examined separately for males and females are presented in Supplemental⁠ Material, Table 3.

**Table 3.** Regression models investigating the effect of recent SLEs on number of overgeneral AMs and specific AMs.

	Overgeneral AM (*n* = 214)						Specific AM (*n* = 214)					
	Model change			Coefficients			Model change			Coefficients		
	*R*^2^	*p*	*f^2^*	*β*	*B* (95% CI)	*p*	*R*^2^	*p*	*f^2^*	*β*	*B* (95% CI)	*p*
**Step 1: Covariates**	**.071**	**.004**	**.077**				**.080**	**.002**	**.087**			** **
Full scale IQ				0.090	0.181 (−0.091, 0.453)	.190				0.177	0.563 (0.137, 0.989)	**.010**
Age				−0.172	−0.316 (−0.562, −0.070)	**.012**				0.195	0.566 (0.181, 0.951)	**.004**
MFQ depressive symptoms				0.198	0.390 (0.123, 0.656)	**.004**				−0.141	−0.438 (−0.856, −0.020)	**.040**
Gender				0.014	0.051 (−0.459, 0.561)	.844				0.005	0.029 (−0.770, 0.828)	.943
**Step 2: Main effect SLEs**	Δ<.001	.836	<.001				Δ<.001	.953	<.001			** **
Recent SLEs				0.016	0.033 (−0.278, 0.344)	.836				−0.005	−0.015 (−0.502, 0.473)	.953
**Step 3: Moderation by gender**	**Δ.044**	**.002**	**.050**				**Δ.044**	**.001**	**.050**			
Recent SLEs x Gender				0.349	0.882 (0.340, 1.423)	**.002**				−0.349	−1.389 (−2.237, −0.540)	**.001**

AM = Autobiographical Memory; CI = Confidence Interval; IQ = Intelligence Quotient; MFQ = Mood and Feelings Questionnaire (Wave 2); SLEs = stressful life events. Results significant at *p* < .05 are indicated in bold. The quadratic term (recent SLEs x recent SLEs) was not significantly associated with overgeneral AM (*β* = 0.245, *B* (95% CI)** = **0.585 (−0.352, 1.522), *R*^2^ change = 0.007, *f*^2^ = 0.007, *p* = .220) or specific AM (*β* = −0.367, *B* (95% CI) = −1.380 (−2.842, 0.082), *R*^2^ change = 0.015, *f*^2^ = 0.017, *p* = .064).

#### Specific autobiographical memory

There was no significant main effect of recent SLEs on specific AMs and there was no evidence for a non-linear relationship (Table 3). However, a significant SLE by gender interaction was also present for specific AMs (*β* = −.349, *B* (95% CI) = −1.389 (−2.237, −.540), *p* = .001). Simple slopes analyses by gender found boys experiencing more recent SLEs recalled *more* specific AMs (*t* = 2.346, *p* = .020), but the association with *fewer* specific AMs in girls was not significant (*t* = −1.862, *p* = .064). Analyses for recent SLEs and positive/negative specific AM in males and females are presented in Supplemental Material,⁠ Table 3.

### Mediation

We next tested whether overgeneral AM for negative cues mediated the association between SLEs and subsequent depression. We focused on overgeneral AM for negative cues given the previously reported association with subsequent DSM-IV depressive symptom count and disorder in this sample. There was no evidence of an indirect effect of overgeneral AM to negative cues on subsequent depression when lifetime SLEs (*b* = .001, SE = .005, 95% CI = −.007, .012) or recent SLEs (*b* < .001, SE = .004, 95% CI = −.009, .010) were predictor variables. There was also no evidence of moderated mediation by gender (lifetime SLEs: *b* = .003, SE = .009, 95% CI = −.013, .026; recent SLEs: *b* = −.011, SE = .016, 95% CI = −.047, .018).

Additional mediation models which were run for completeness also found no evidence for mediation or mediated moderation of the other AM indices (Supplemental Material, Table 4).

### Sensitivity analyses

Sensitivity analyses separated the more severe (e.g., death of parent, brother or sister) and less severe (e.g., mother/father losing job) lifetime SLEs (see Supplemental Material, Table 1) to assess the role of event severity in the unexpected observation that *greater* lifetime SLEs were associated with a *greater* number of specific AMs. More severe lifetime SLEs significantly predicted total specific AMs (*β* = .144, *B* (95% CI) = .410 (.025, .794), *p* = .037), specific AMs cued with positive words (*β* = .197, *B* (95% CI) = .314 (.102, .527), *p* = .004) and specific positive memories (*β* = .239, *B* (95% CI) = .384 (.170, .597), *p* < .001). Less severe lifetime SLEs were not associated with specific AMs (*β* = .131, *B* (95% CI) = .381 (−.016, .777), *p* = .060), but was significantly associated with specific AMs for positive cues (*β* = .150, *B* (95% CI) = .245 (.024, .466), *p* = .030) and specific positive memories (*β* = .141, *B* (95% CI) = .232 (.008, .455), *p* = .042).

To determine whether results were attributable to a prior depressive episode, analyses assessing the relationship between SLEs and AM were repeated excluding individuals with a prior depressive episode. All results remained the same (results available from first author) with one exception - simple slopes analysis revealed males with more recent SLEs reported fewer overgeneral AMs and more specific AMs but the positive association between recent SLEs and overgeneral AM in girls was no longer significant (*t* = 1.860, *p* = .064). Consequently, presence of a prior depressive episode is unlikely to affect results.

## Discussion

The current study investigated the relationship between lifetime and recent SLEs and AM in an adolescent sample at high familial risk for MDD. No main effect of lifetime SLEs on overgeneral AM was observed. Unexpectedly, a greater number of lifetime SLEs was associated with recall of more specific AMs. For recent SLEs, results differed: there were no main effects on AM but there were significant interactions with gender. For boys, a greater number of recent SLEs was associated with *fewer* overgeneral AMs and *more* specific AMs. For girls, the effect was in the opposite but expected direction such that more recent SLEs were associated with more overgeneral AMs. However, there was no evidence that AM mediated the relationship between SLEs and subsequent DSM-IV depressive symptom count or for moderated mediation by gender. These results suggest that the relationship between SLEs and AM is complex, differing based on event recency, gender, and overgenerality/specificity. Results also suggest that SLEs and AM may exert independent effects on subsequent depression.

The present study found no association between lifetime SLEs and overgeneral AM but a greater number of lifetime SLEs were associated with more specific AMs. This pattern of results was unexpected given that previous literature has demonstrated that a greater number of SLEs and overgeneral AMs are associated with increased risk for MDD and greater depressive symptoms (Kendler et al., 1999; Liu et al., 2013; Rawal & Rice, 2012a; Williams et al., 2007) and that more traumatic events have been implicated in overgeneral AM (Williams et al., 2007). One possible explanation for the unexpected finding is that participants who recall more lifetime SLEs may simply have better memory in general. Previous research in adults has highlighted that difficulty maintaining task goals or instructions in working memory is associated with greater overgeneral AM (Yanes, Roberts, & Carlos, 2008), so better recall of task instructions could underlie our increased specificity for lifetime SLEs finding. However, this seems unlikely to be the case as more lifetime SLEs were associated with lower IQ and poorer working memory (Table 1). Sensitivity analyses also suggest this result is unlikely to be due to a past episode of MDD.

Research has highlighted the mood enhancing benefits of recalling positive AMs (Joormann et al., 2007; Ramirez et al., 2015). It is therefore possible that recalling specific positive AMs may act as a coping strategy in the presence of previous SLEs. Indeed, moderate levels of stress may be beneficial and buffer against depressive episodes (Boyce & Ellis, 2005; Ellis & Boyce, 2008; Shapero et al., 2015). Although the association for lifetime SLEs and specific AMs was stronger for positive cues and memories, overlapping CIs suggest relationships with valence did not differ significantly. Furthermore, AM specificity to positive cues did not mediate the relationship between lifetime SLEs and subsequent depression symptoms (Supplemental Material, Table 4) so recalling positive AMs in response to SLEs is unlikely to act as a coping strategy.

By definition, the measure of SLEs examined in this study was broad and included severe but less common events (e.g., death of a parent) as well as less severe, more common events (e.g., mother or father losing job, death of a pet). All SLEs were rated as having a negative impact by combined parent and child reports (Supplemental Material, Table 1) and associations with AM specificity indices remained when more severe and less severe SLEs were examined separately. Correlations of lifetime SLEs with demographic and psychopathology variables were consistent with what would be predicted and existing literature (i.e., more SLEs were associated with lower IQ, poverty, older age and greater current and subsequent depressive symptomatology) (Franz et al., 2011; Goodyer et al., 1993; Hartlage, Alloy, Vazquez, & Dykman, 1993; McLeod & Kessler, 1990; Repetti et al., 2002), but there may be inherent differences between traumatic events and SLEs. Trauma involves witnessing or experiencing an incident that may be life-threatening (e.g., road traffic accident, sexual assault) whereas SLEs (e.g., parental divorce, relative dying from natural causes) are less likely to involve threat to life. Consequently, the unexpected results for lifetime SLEs and AM specificity compared to the trauma and AM literature (Moore & Zoellner, 2007; Ono et al., 2015) may be a result of differences between SLEs and trauma.

Gender differences were observed for associations between recent SLEs on overgeneral and specific AM: more SLEs were associated with more overgeneral AMs in girls (the hypothesised direction) and boys displayed increased SLEs with decreased overgeneral AM and more specific AMs (the opposite direction). Such results could indicate that boys and girls react differently to recent SLEs and there is some evidence to support this view. First, girls are more sensitive to social stressors compared to boys and are more likely to develop depressive symptoms following social stress (Calvete, Camara, Estevez, & Villardon, 2011; Oldehinkel & Bouma, 2011; Shih et al., 2006). Second, a previous analysis of this sample illustrated that the association between overgeneral AM and subsequent depressive symptoms and disorder differed significantly by gender such that this relationship was substantially stronger for girls than boys (Rawal & Rice, 2012a). Our moderated mediation analysis suggests that overgeneral AM for negative cues is not a mechanism by which the stronger relationship between recent SLEs and MDD in adolescent girls compared to boys can be explained. One possibility is that boys and girls differ in how and what they recall; for example, research has shown that females’ AMs contain more emotional and relational content (Fivush, 2011) and that there are gender differences in brain activity for the emotional aspects of AM (Compere et al., 2016). Nevertheless, no mean-level differences in AM were observed between genders (Table 1; Supplemental Material, Table 2). Alternatively, as girls experienced more recent SLEs, this could have affected the results if a certain number of SLEs was required to produce the anticipated overgeneral AM response (i.e., a threshold effect). Nevertheless, results of analyses using quadratic terms for recent SLEs suggest this is unlikely to be the case. Taken together, our results point to overgeneral AM and SLEs having independent effects on MDD and these effects varying by gender.

In boys and girls, associations between recent SLEs and AM valence were sometimes observed more strongly for one valence than the other (Supplemental Material, Table 3). As overgeneral AM is thought to develop in response to functional avoidance of negative stimuli (Williams et al., 2007) and previous analyses of this sample found negative overgeneral AM to predict MDD (Rawal & Rice, 2012a), it was anticipated that more SLEs would be associated with increases in overgeneral responses to negative cues and negative overgeneral memories. However, in current analyses, observed stronger associations for negative cues compared to positive cues did not translate to significant differences in valence associations as CIs overlapped. Nevertheless, assessment of SLEs and AM valence in younger samples is warranted to determine if significant valence differences are apparent earlier in development.

If overgeneral AM develops via a functional avoidance mechanism it may mediate the relationship between SLEs and subsequent depression. However, overgeneral AM to negative cue words did not mediate the relationship between lifetime or recent SLEs and future depression, and there was no evidence of moderated mediation by gender. Given that we find recent SLEs are associated with increased overgeneral AM (negative) in girls and previous analysis of this sample has found overgeneral AM to negative cues is associated with prospective increases in depressive symptoms and new onset MDD (Rawal & Rice, 2012a), this pattern of results suggests that SLEs and overgeneral AM to negative cues exert independent effects on future depression. Previous work has mainly focused on overgeneral AM as a moderator of the SLE-MDD relationship with mixed findings (Anderson et al., 2010; Crane et al., 2016; Gibbs & Rude, 2004; Sumner et al., 2011). Our work extends developmental findings and highlights that the relationship between overgeneral AM to negative cues and future MDD is unlikely to develop as a result of recent or lifetime SLEs. Nevertheless, more complex relationships between overgeneral AM and subsequent depression may also exist; for instance, moderation by socioeconomic deprivation and other cognitive liability factors such as rumination (Hamlat et al., 2015; Stange, Hamlat, Hamilton, Abramson, & Alloy, 2013).

It should be noted that the sample was at high risk of MDD from having at least one parent with recurrent depression which may have affected results. Having a parent with a mental health condition may in itself be a SLE (Chapman et al., 2004; Cheong, Sinnott, Dahly, & Kearney, 2017; Hammen, 2002; Hammen, Shih, & Brennan, 2004). Furthermore, offspring of depressed parents tend to report more SLEs than the general population (Bouma, Ormel, Verhulst, & Oldehinkel, 2008; Goodman & Gotlib, 1999). Consequently our sample is likely to be enriched for SLEs but this estimate does not include parent mental health as a SLE. In addition, as parents themselves likely have increased levels of overgeneral AM (Williams et al., 2007), their less elaborative reminiscing may contribute to their offspring developing less elaborated (or less specific) AMs (Fivush, Haden, & Reese, 2006). Thus, our sample may be enriched for overgeneral AM and reduced AM specificity. Although having such a high-risk group is informative, replication in a community-based/population sample is required to determine whether current results are specific to this high-risk sample.

Findings also highlight the difference between overgeneral AM and AM specificity in that different patterns of association with SLEs, depressive symptomatology and demographic correlates were observed for overgeneral and specific AM. For instance, number of lifetime SLEs was associated with AM specificity but not overgeneral AM. Moreover, specific and overgeneral AM also appeared to have different prospective relationships with DSM-IV depressive symptom count and MDD (based on the current study and previous work with this sample (Rawal & Rice, 2012a)). In combination, these findings suggest that AM specificity and overgeneral AM are not different ends of the same spectrum in this sample.

### Strengths and limitations

This study benefits from longitudinal data from an informative sample. Exploration in high-risk individuals is merited due to the importance of this group for targeted prevention approaches. The use of longitudinal data allows exploration of SLEs and MDD across development. Measures of SLEs and depressive symptoms incorporated reports from multiple informants. This approach is recommended in routine clinical practice for depression in young people and is also less susceptible to reporting bias than information from single informants (Kessler, 1997; Rutter & Sroufe, 2000). This study also examined SLEs experienced both previously in the lifetime and recently, a practice rarely used in the literature.

Nevertheless, this study should be viewed in light of a number of limitations. First, SLEs were reported retrospectively. This may have been particularly problematic for lifetime SLEs which required retrospective recall across the lifetime as opposed to during the previous 12 months for recent SLEs. To some degree, collating reports across multiple informants assuages this limitation (Gest et al., 1999). Second, life events were assessed by questionnaire checklist rather than interview. Third, the analysis was conducted in a high-risk group and may not generalise to low-risk individuals. Finally, memory content valence was rated by researcher as no participant ratings were available. Consequently, participants may have experienced emotion of the memory differently to researcher rating.

## Conclusion

This research has begun to untangle the complex relationship between SLEs and AM in a sample at high risk of MDD. Research into overgeneral AM is important as it may act as a cognitive vulnerability factor for MDD. The present study found a main effect of lifetime SLEs on AM specificity. A gender difference was also found which suggests that following exposure to recent SLEs, boys and girls react differently as far as overgeneral AM is concerned. AM did not mediate the relationship between SLEs and subsequent DSM-IV depressive symptom count. In sum, although SLEs and overgeneral AM are well-established risk factors for MDD, they may exert independent effects in adolescents at high familial risk of depression.

## Supplementary Material

Supplemental Material
