# Supplementary material for: Examining the relationship between stressful life events and overgeneral autobiographical memory in adolescents at high familial risk of depression
Source: Memory. 2018 Aug 18;27(3):314–27. doi: 10.1080/09658211.2018.1508591 (PMC6343111; doi:10.1080/09658211.2018.1508591)
Supplement: Supplemental Material [file PMEM_A_1508591_SM1068.docx]

Supplemental Table 1. Frequency and average impact of lifetime and recent stressful life events

|  | **Stressful life event (SLE)** | **N** | **Frequency (%)** | **Mean impact** | **SD** |
| --- | --- | --- | --- | --- | --- |
| ***Lifetime*** | |  |  |  |  |
|  | Death of parent, brother or sister* | 11 | 4.280 | 4.682 | 0.560 |
|  | Death of a close friend* | 19 | 7.393 | 4.632 | 0.523 |
|  | Serious illness or injury to child* | 69 | 26.848 | 4.543 | 0.741 |
|  | Bullying by another young person | 130 | 50.584 | 4.538 | 0.566 |
|  | Serious injury or illness to parent, brother or sister* | 122 | 47.471 | 4.475 | 0.589 |
|  | Serious illness or injury to a close friend | 33 | 12.840 | 4.439 | 0.659 |
|  | Death of grandparent | 137 | 53.307 | 4.372 | 0.683 |
|  | Death of pet | 164 | 63.813 | 4.372 | 0.640 |
|  | Parent going to prison* | 3 | 1.167 | 4.333 | 0.577 |
|  | Parents divorced or separated* | 91 | 35.409 | 4.220 | 0.810 |
|  | Parent getting into trouble with the police | 20 | 7.782 | 3.875 | 0.759 |
|  | Father losing job | 42 | 16.342 | 3.750 | 0.828 |
|  | Mother losing job | 24 | 9.339 | 3.708 | 0.859 |
| ***Recent (in the past 12 months)*** | |  |  |  |  |
|  | Death of parent, brother or sister | 1 | 0.389 | 5.000 | 0.000 |
|  | Parent getting into trouble with the police | 2 | 0.778 | 5.000 | 0.000 |
|  | Parent going to prison | 1 | 0.389 | 5.000 | 0.000 |
|  | Death of a close friend | 15 | 5.837 | 4.733 | 0.458 |
|  | Parents divorced or separated | 8 | 3.113 | 4.625 | 0.518 |
|  | Serious illness or injury to a close friend | 15 | 5.837 | 4.567 | 0.495 |
|  | Bullying by another young person | 39 | 15.175 | 4.526 | 0.769 |
|  | Death of grandparent | 35 | 13.619 | 4.486 | 0.562 |
|  | Death of pet | 59 | 22.957 | 4.441 | 0.595 |
|  | Losing a close friend through arguments or being dropped | 49 | 19.066 | 4.429 | 0.645 |
|  | Serious injury or illness to parent, brother or sister | 37 | 14.397 | 4.419 | 0.618 |
|  | Increased quarrelling between parents | 53 | 20.623 | 4.311 | 0.630 |
|  | Doing badly in an exam | 65 | 25.292 | 4.238 | 0.607 |
|  | Close friend moves a long way away | 28 | 10.895 | 4.214 | 0.947 |
|  | Doing badly in school work | 46 | 17.899 | 4.152 | 0.613 |
|  | Breaking up with a boyfriend/girlfriend | 63 | 24.514 | 4.119 | 0.776 |
|  | Parent nagging or picking on this child more | 63 | 24.514 | 4.095 | 0.812 |
|  | Serious illness or injury to child | 9 | 3.502 | 4.056 | 1.286 |
|  | Parent less interested or loving towards child | 21 | 8.171 | 4.024 | 0.782 |
|  | Father losing job | 27 | 10.506 | 3.685 | 1.011 |
|  | Mother losing job | 10 | 3.891 | 3.600 | 0.937 |

Impact severity on a scale of 1 to 5 (1 Very pleasant, 2 A bit pleasant, 3 No effect/neutral, 4 A bit unpleasant, 5 Very unpleasant). SLE = stressful life event. * indicates more severe stressful life event in sensitivity analyses.

Supplemental Table 2. Descriptive information and associations between SLEs, AM, depressive symptoms, covariates and descriptive variables for boys (above the diagonal) and girls (below the diagonal).

|  | 1 | 2 | 3 | 4 | 5 | 6 | 7 | 8 | 9 | 10 | 11 | 12 | 13 | 14 | 15 | 16 |
| --- | --- | --- | --- | --- | --- | --- | --- | --- | --- | --- | --- | --- | --- | --- | --- | --- |
| 1. Age |  | -.024 | .018 | .041 | -.173 | .051 | -.065 | .027 | .192 | -.066 | -.126 | .097 | -.109 | -.094 | .126 | .049 |
| 2. IQ | **-.195** |  | **.690** | **-.320** | -.072 | -.105 | -.158 | **-.226** | -.150 | .004 | -.065 | **.259** | -.052 | -.052 | **.215** | **.245** |
| 3. Working memory | -.049 | **.653** |  | -.148 | -.080 | -.055 | -.057 | -.059 | **-.244** | .109 | -.176 | **.241** | -.132 | -.151 | **.224** | **.204** |
| 4. Economic disadvantage | .079 | **-.195** | -.066 |  | .170 | .059 | .099 | .037 | **.215** | .120 | -.077 | -.038 | -.056 | -.068 | -.072 | .002 |
| 5. W2 depressive symptoms (MFQ) | **.229** | -.118 | -.100 | **.173** |  | **.649** | **.666** | **.380** | **.239** | **.484** | .056 | -.013 | -.147 | **.246** | -.003 | -.020 |
| 6. W3 depressive symptoms (MFQ) | .074 | -.019 | .035 | .143 | **.610** |  | **.523** | **.600** | .147 | **.334** | -.027 | .092 | -.151 | .117 | .087 | .077 |
| 7. W2 DSM-IV depressive symptom count (CAPA) | **.362** | **-.192** | **-.191** | **.191** | **.689** | **.451** |  | **.589** | **.225** | **.526** | -.041 | .002 | -.115 | .054 | .022 | -.018 |
| 8. W3 DSM-IV depressive symptom count (CAPA) | **.244** | -.089 | -.075 | **.230** | **.548** | **.713** | **.613** |  | .039 | **.410** | -.029 | -.018 | -.080 | .038 | -.029 | -.003 |
| 9. SLEs (lifetime) | **.335** | **-.193** | **-.194** | **.240** | **.193** | .077 | **.214** | **.243** |  | .095 | -.058 | .100 | -.142 | .053 | .141 | .041 |
| 10. SLEs (recent) | .154 | -.104 | -.131 | -.105 | **.552** | **.331** | **.412** | **.314** | .165 |  | -.160 | .178 | -.122 | -.130 | .111 | .201 |
| 11. Overgeneral AM (total) | -.081 | .107 | .092 | .074 | **.237** | **.235** | .114 | **.207** | -.064 | **.216** |  | **-.523** | **.814** | **.793** | **-.507** | **-.426** |
| 12. Specific AM (total) | .113 | .155 | .019 | -.079 | -.163 | **-.190** | -.092 | -.139 | .143 | **-.212** | **-.495** |  | **-.437** | **-.403** | **.880** | **.896** |
| 13. Overgeneral AM (positive cues) | -.079 | .073 | .041 | -.050 | **.182** | **.188** | .074 | .087 | -.077 | .125 | **.819** | **-.440** |  | **.292** | **-.538** | **-.249** |
| 14. Overgeneral AM (negative cues) | -.054 | .103 | .109 | **.172** | **.211** | **.203** | .110 | **.246** | -.028 | **.235** | **.820** | **-.372** | **.344** |  | **-.271** | **-.441** |
| 15. Specific AM (positive cues) | .097 | **.202** | .095 | .024 | **-.179** | **-.176** | -.101 | -.104 | **.182** | **-.240** | **-.441** | **.872** | **-.527** | **-.195** |  | **.579** |
| 16. Specific AM (negative cues) | .101 | .072 | -.058 | **-.160** | -.106 | -.157 | -.060 | -.136 | .069 | -.134 | **-.427** | **.879** | **-.247** | **-.454** | **.534** |  |
| Boys Mean | 13.500 | 95.010 | 95.853 |  | 14.061 | 12.561 | 1.530 | 1.343 | 3.670 | 2.437 | 1.961 | 6.755 | 1.059 | 0.902 | 3.451 | 3.304 |
| Boys SD | 1.887 | 10.446 | 13.694 |  | 13.146 | 11.600 | 1.708 | 1.472 | 1.995 | 1.951 | 1.665 | 2.830 | 1.061 | 1.010 | 1.539 | 1.646 |
| Girls Mean | 13.890 | 97.546 | 98.632 |  | 18.688 | 18.386 | 2.150 | 2.111 | 3.838 | 3.076 | 2.090 | 6.858 | 1.142 | 0.948 | 3.561 | 3.297 |
| Girls SD | 2.094 | 12.438 | 13.741 |  | 13.695 | 14.650 | 2.077 | 2.123 | 1.902 | 2.160 | 1.965 | 2.981 | 1.198 | 1.200 | 1.679 | 1.725 |

AM = autobiographical memory; CAPA = Child and Adolescent Psychiatric Assessment; IQ = Intelligence Quotient; MFQ = Mood and Feelings Questionnaire; SLEs = stressful life events; W2 = Wave 2; W3 = Wave 3. Significant correlations indicated in bold.

Supplemental Table 3. Association between recent SLEs and overgeneral AMs and specific AMs by cue/memory valence and gender.

|  | |  | **Overgeneral AM** | | | | | | **Specific AM** | | | | | |
| --- | --- | --- | --- | --- | --- | --- | --- | --- | --- | --- | --- | --- | --- | --- |
|  | |  | **Boys** | | | **Girls** | | | **Boys** | | | **Girls** | | |
|  | | | **β** | **bootstrapped**  **95% CI** | **p** | **β** | **bootstrapped**  **95% CI** | **p** | **β** | **bootstrapped**  **95% CI** | **p** | **β** | **bootstrapped**  **95% CI** | **p** |
| **Cue valence** | | |  |  |  |  |  |  |  |  |  |  |  |  |
|  | *Positive* | | -0.057 | -0.312, -0.223 | .644 | 0.007 | -0.218, 0.205 | .945 | 0.151 | -0.104, 0.405 | .212 | -0.206 | -0.413, 0.038 | **.043** |
|  | *Negative* | | -0.295 | -0.571, -0.005, | **.015** | 0.230 | 0.068, 0.454 | .**024** | 0.268 | 0.078, 0.497 | **.025** | -0.084 | -0.300, 0.144 | .424 |
| **Memory valence** | | |  |  |  |  |  |  |  |  |  |  |  |  |
|  | *Positive* | | -0.108 | -0.366, 0.158 | .374 | 0.022 | -0.196, 0.201 | .838 | 0.176 | -0.070, 0.409 | .146 | -0.181 | -0.395, 0.019 | .078 |
|  | *Negative* | | -0.215 | -0.464, 0.080 | .081 | 0.224 | 0.041, 0.444 | **.023** | 0.212 | -0.017, 0.466 | .084 | -0.156 | -0.366, 0.047 | .138 |

AM = Autobiographical Memory; CI = Confidence Interval. Significant results are indicated in bold.

|  |  | **Mediation** | | | | | | | | | | | | | **Moderated mediation** | | |
| --- | --- | --- | --- | --- | --- | --- | --- | --- | --- | --- | --- | --- | --- | --- | --- | --- | --- |
| **Predictor** | **Mediator** | **Effect of X on M** | | | **Effect of M on Y** | | | | **Direct Effect** | | | | **Indirect Effect** | | **Index of Moderated Mediation** | | |
| **(X)** | **(M)** | **b (SE)** | **95% CI** | **p** | | **b (SE)** | **95% CI** | **p** | | **b (SE)** | **95% CI** | **p** | **b (SE)** | **95% CI** | | **b (SE)** | **95% CI** |
| Lifetime SLEs | Overgeneral AM (negative) | -0.034 (0.038) | -0.110, 0.042 | .377 | | -0.038 (0.075) | -0.185, 0.109 | .611 | | 0.042 (0.041) | -0.039, 0.122 | .307 | 0.001 (0.005) | -0.007, 0.012 | | 0.003 (0.009) | -0.013, 0.026 |
| (n=205) | Overgeneral AM (positive) | -0.063 (0.040) | -0.141, 0.016 | .119 | | -0.019 (0.072) | -0.160, 0.123 | .794 | | 0.042 (0.041) | -0.039, 0.122 | .308 | 0.001 (0.005) | -0.011, 0.012 | | -0.001 (0.006) | -0.013, 0.013 |
|  | Specific AM (negative) | 0.078 (0.061) | -0.042, 0.199 | .200 | | -0.043 (0.047) | -0.136, 0.049 | .357 | | 0.046 (0.041) | -0.034, 0.127 | .257 | -0.003 (0.005) | -0.017, 0.004 | | -0.003 (0.008) | -0.024, 0.009 |
|  | Specific AM (positive) | **0.176 (0.058)** | **0.062, 0.289** | **.003** | | -0.096 (0.049) | -0.194, 0.001 | .052 | | 0.060 (0.041) | -0.021, 0.141 | .148 | -0.017 (0.010) | -0.039, 0.001 | | -0.007 (0.012) | -0.032, 0.018 |
| Recent SLEs | Overgeneral AM (negative) | 0.007 (0.042) | -0.075, 0.090 | .860 | | -0.570 (0.077) | -0.209, 0.096 | .462 | | 0.040 (0.046) | -0.052, 0.131 | .391 | <0.001 (0.004) | -0.009, 0.010 | | -0.011 (0.016) | -0.047, 0.018 |
| (n=207) | Overgeneral AM (positive) | 0.029 (0.044) | -0.058, 0.117 | .508 | | -0.063 (0.073) | -0.207, 0.081 | .392 | | 0.041 (0.046) | -0.050, 0.133 | .374 | -0.002 (0.005) | -0.015, 0.007 | | -0.010 (0.013) | -0.041, 0.011 |
|  | Specific AM (negative) | 0.051 (0.065) | -0.077, 0.178 | .433 | | -0.034 (0.050) | -0.134, 0.065 | .496 | | 0.041 (0.046) | -0.050, 0.133 | .376 | -0.002 (0.004) | -0.013, 0.005 | | 0.011 (0.014) | -0.017, 0.040 |
|  | Specific AM (positive) | -0.059 (0.062) | -0.182, 0.064 | .343 | | -0.039 (0.052) | -0.142, 0.064 | .459 | | 0.037 (0.047) | -0.054, 0.129 | .425 | 0.002 (0.005) | -0.007, 0.013 | | 0.011 (0.015) | -0.018, 0.044 |

Supplemental Table 4. Summary of mediation models testing for indirect effect of stressful life events on depression symptoms through autobiographical memory indices.

Mediation models controlling for current depressive symptoms on the Mood and Feelings Questionnaire (Wave 2). Significant results are indicated in bold.
